# RGDSP-functionalized peptide hydrogel stimulates growth factor secretion via integrin αv/PI3K/AKT axis for improved wound healing by human amniotic mesenchymal stem cells

**DOI:** 10.3389/fbioe.2024.1385931

**Published:** 2024-10-14

**Authors:** Wei Wei, Lei Huang, Luoying Chen, Huanhuan He, Yanfei Liu, Yuan Feng, Fengqin Lin, Hui Chen, Qing He, Junhong Zhao, Haihong Li

**Affiliations:** ^1^ Key Laboratory of Cell Engineering of Guizhou Province, Affiliated Hospital of Zunyi Medical University, Zunyi, China; ^2^ The Clinical Stem Cell Research Institute, Affiliated Hospital of Zunyi Medical University, Zunyi, China; ^3^ Department of Wound Repair and Dermatologic Surgery, Taihe Hospital, Hubei University of Medicine, Shiyan, China

**Keywords:** self-assembling peptide hydrogel, human amniotic mesenchymal stem cells, integrin, paracrine function, PI3K/AKT, wound healing

## Abstract

The wound healing process involves communication among growth factors, cytokines, signaling pathways, and cells in the extracellular matrix, with growth factors acting as key regulators. Although stem cells can promote wound healing by secreting diverse growth factors, their therapeutic potential is hindered by poor survival and engraftment. Mimicking the stem cell-matrix interactions can improve stem cell survival, regulate their fate, and even enhance their paracrine effects. This study investigated the use of composite RGDmix hydrogel, which can support the survival and proliferation of human amniotic mesenchymal stem cells (hAMSCs), and effectively increase the expression of various growth factors, thereby promoting wound re-epithelialization, angiogenesis, and epidermal maturation. At last, the specific role of integrin αv and PI3K/AKT signaling pathways in the secretion of growth factors were examined by silencing them *in vitro* and *in vivo*. Results suggested that the RGDmix hydrogel improved the secretion of growth factors by hAMSCs through the RGDSP/integrin αv/PI3K/AKT axis, thereby enhancing the therapeutic effect in wound healing.

## 1 Introduction

Skin wound healing is a complex multi-stage process involving a cascade of molecular and cellular events. The three stages of acute inflammation, cell proliferation, and remodeling overlap with each other to repair the injury (Kaur et al., 2022). Biophysical and biochemical cues from the extracellular matrix (ECM) have been characterized that regulate distinct pathways during wound regeneration (Bakhshandeh et al., 2022). The ideal scenario in wound healing involves the complete restoration of defective tissue by tissue cells with identical structure and function, stemming from the same origin (Nilforoushzadeh et al., 2022).

In regenerative medicine, mesenchymal stem cells (MSCs) have gained extensive attention for their ability to promote tissue regeneration. This is attributed to their capacity to secrete a wide range of growth factors in response to the surrounding microenvironment. These soluble factors have the capability to influence neighboring cells through paracrine signaling, thereby facilitating processes such as angiogenesis, anti-inflammatory responses, proliferation, and migration. This paracrine signaling ultimately serves to enhance the overall wound-healing process. However, 95% of the administered MSCs lose their activity within a few hours after transplantation into the damaged tissue (Wechsler et al., 2021). The detrimental host microenvironment (Mitrousis et al., 2018) and mechanical disruption of cells during the injection process (Dromel et al., 2020) are major contributing factors to the low cell retention rates.

Increasing evidence shows that the natural extracellular matrix (ECM) environment plays a crucial role in regulating the behavior, fate, and viability of stem cells (Nicolas et al., 2020). A suitable microenvironment, which effectively sustains the viability and bioactivity of transplanted MSCs, is essential for the wound healing. Thereby, researchers are exploring new delivery strategies, such as bioengineered hydrogels, to improve MSCs survival and function. Injectable hydrogels have been extensively investigated for improving MSCs activities, survival and therapeutic efficacy. These hydrogels show great potential due to their biocompatibility, injectability, biodegradability, and high permeability for growth factors and cell metabolites (Guan et al., 2022). Despite numerous attempts to engineer hydrogels to mimic the properties of native ECM, the incorporation of cell-adhesive peptides into hydrogels can promote the survival of MSCs, manipulate their fate, and enhance their paracrine activity. Our previous study has shown that the incorporation of adhesive peptides, such as RGDSP, TTSWSQ and GFOGER, is in favor of maintaining the phenotypic characterization and promoting the pluripotent markers expression of hAMSCs. Regulation of hAMSCs may be mediated by distinct integrin-mediated pathways (Zhang et al., 2021). These findings could aid in maximizing the therapeutic potential of hAMSCs for wound healing. Although various hydrogels incorporate short peptides to mimic the cell-ECM interaction, it is still unclear whether these peptides can effectively promote growth factor secretion and enhance the therapeutic efficacy of hAMSCs in wound healing, as well as their underlying molecular mechanisms.

In this study, we utilized the composite hydrogel RGDmix (RADA16-RGDSP: RADA16 = 7:3) as a synthetic ECM mimic to encapsulate and deliver hAMSCs. In a murine model of excisional wound healing, we investigated the effects of incorporating the RGDSP ligand on hAMSCs growth factor secretion, and therapeutic efficiency. Results show that the addition of RGDSP to the hydrogel promotes hAMSCs growth factors secretion, by enhancing the integrin αv/PI3K/AKT axis, ultimately accelerating the wound healing process. Further research is required to gain a better understanding of the impact and mechanisms of the microenvironment on the secretome of MSCs and to develop biomaterials that provide optimized biophysical and biochemical cues to guide MSC secretome behavior that can lead to enhanced clinical translation outcomes.

## 2 Materials and methods

### 2.1 Materials

RADA16 and RADA16-RGDSP (Ac-RADARADARADARADAGGRGDSP-CONH_2_) were synthesized by SciLight Biotechnology LLC. (China). The peptide purities were greater than 95%. The peptides were dissolved with autoclaved deionized water to 20 mg/mL (2%) as stock solutions. For RGDmix, the RADA-RGDSP solution was mixed with RADA16 solution at a ratio of 7:3. Subsequently, the mixture underwent sonication for a duration of 20 min to disrupt the molecular interaction. Following sonication, the mixed solution was left at room temperature for a minimum of 24 h and subsequently stored at a temperature of 4°C.

### 2.2 Isolation and characterization of hAMSCs

Human placentas were collected following caesarean section procedures performed on healthy women who tested negative for HIV-I, cytomegalovirus, syphilis, and hepatitis virus B and C. Legally informed consent ofeach participant or their legally authorized representative was obtained before the procedure began. Approval for the research protocol was granted after a comprehensive evaluation by the medical ethics committee at Zunyi Medical University (ZMUER 2018-1-154).

The hAMSCs were isolated and cultured according to a reported method (Zhang et al., 2021). Cells from passage 3 were used in all experiments. In brief, hAMSCs were isolated from the amniotic membrane of the human placentas using a pancreatin and collagenase digestion method as described previously. Upon isolation, the hAMSCs were cultivated in low glucose DMEM (Gibco-BRL) with the addition of 1% glutaMAX (Gibco-BRL), 10% fetal bovine serum (Ausbian), and a concentration of 10 ng/mL of human bFGF (Sigma-Aldrich). The cell culture was maintained in a 5% CO_2_ incubator.

To characterize the hAMSCs, a cellular suspension underwent incubation with a panel of antibodies, including CD73-APC, CD90-FITC, CD105-PerCP-Cy5.5, as well as PE-conjugated anti-CD19, CD34, CD45, CD11b, and HLA-DR antibodies from BD Biosciences, along with their respective isotype controls. The BD Biosciences FACSCalibur flow cytometry system was used to analyze the specific surface antigen phenotypes.

To evaluate the trilineage differentiation potential of hAMSCs, we followed established protocols for inducing osteogenic, chondrogenic, and adipogenic lineages (Naeem et al., 2022). Briefly, hAMSCs were cultured in lineage-specific differentiation media (Cyagen Biosciences, China). Subsequently, the cells were fixed in 4% paraformaldehyde. Staining with Alizarin Red S, Toluidine Blue, and Oil Red O solutions was performed to assess osteogenic, chondrogenic, and adipogenic differentiation, respectively. Immunohistochemistry assays were conducted to analyze the expression of Vimentin (Sigma-Aldrich) and cytokeratin 19 (CK19, Gene Tech) in hAMSCs. Color development was achieved by treating the samples with diaminobenzidine for 30 s, followed by nuclear counterstaining using hematoxylin.

### 2.3 Encapsulation of hAMSCs on self-assembling peptide scaffolds

The characterized hAMSCs at passage 3 subjected to trypsin digestion and subsequently resuspended (5 × 10^5^ cells/mL). The cell suspensions were carefully mixed with equal volumes of 1% RADA16 or RGDmix solutions, and then, 200 μL of the mixtures was carefully added into the center of the wells of a 24-well plate. Then a gentle addition of additional medium was conducted to trigger the hydrogelation of the peptides. The medium was changed three times in the first 2 h to balance the pH of the hydrogels.

### 2.4 Cell viability in the hydrogels

To study the viability of hAMSCs cultured in the hydrogels, 50 μL of the above mentioned hydrogel-cell mixtures was added into the wells of a 96-well plate. After 4 days of culture, the hAMSCs were rinsed and labeled by a live/dead kit (Invitrogen, United States). The cellular morphology within the hydrogels was examined using optical microscopy. Meanwhile, 5 × 10^3^ cells were cultured on a petri dish as the 2D control group.

Cell proliferation was evaluated using the Cell Counting Kit-8 (CCK-8) assay, with absorbance measurements at 450 nm taken using the Thermo Scientific Multiskan GO.

To assess hAMSCs adhesion on hydrogels, 100 μL of peptide solution was uniformly coated on the wells of a 96-well plate, resulting in the formation of hydrogel membranes with a thickness of approximately 2 mm. The plate was then incubated at 37°C for 2 h to facilitate hydrogel formation. Subsequently, 3,000 cells were seeded onto each hydrogel-coated well and incubated for 1 and 3 h, respectively. Following incubation, non-adherent and loosely adherent hAMSCs were gently washed away with PBS, while the remaining adherent cells were harvested and subjected to CCK-8 assay for detection. All experimental points were replicated using three plates.

### 2.5 Skin wound model and treatment

C57BL/6 mice were purchased from Speyfo Biotechnology Co., Ltd. (SCXK Beijing 2016-0002) at the age of 6–8 weeks with a body weight of 20–25 g. The animal experimental procedures followed the animal management regulations established by Zunyi Medical University (ZMUER2018-2-149). This study also fully adhered to the principles of the 3Rs to minimize animal suffering and ensure ethical practices.

Briefly, the C57BL/6 mice were anesthetized and disinfected with 75% alcohol after shaving the hair. A full-thickness skin wound with an 8 mm diameter was created on the dorsal region of each mouse. Rubber rings with an inner diameter of 8 mm were firmly attached to the edge of the skin surrounding the wound beds. To verify the efficacy, mice were randomly assigned to five groups: control group (serum-free medium-treated only), hAMSCs-treated group, RGDmix-treated group, RADA16 + hAMSCs co-treated group, and RGDmix + hAMSCs co-treated group. In the hAMSC-treated group, 100 μL of a serum-free cell suspension (2 × 10^7^ cells/mL) was injected into the wound surface. In the co-treated groups, The hydrogel/hAMSC constructs were created by combining 2% peptides with the hAMSC suspension (4 × 10^7^ cells/mL) in a 1:1 ratio. Then, 100 μL of the construct was injected into the wound bed after surgical excision of the skin. After treatment, Tegaderm dressings (3M, United States) were utilized to cover all the wounds.

At 7, 14, and 21 days after injury, the swelling, color, secretion, wound size, and granulation tissue growth were observed, and photographed (n = 5). After quantifying the wound area, the wound healing rates were computed. At day 21, the quantification of hair follicles and measurement of epidermal thickness were performed.

The excised wound sites were immersed in a 4% paraformaldehyde solution for overnight fixation and paraffin embedded for further investigation. Histological changes and the degree of collagen maturity were observed using tissue sections stained with HE and Masson’s trichrome. Immunofluorescence staining of sections was performed using primary antibodies against Ki67 (Abcam, United Nations), CD31 (Abcam, United Nations), Anti-Human Nuclear Antigen antibody (Abcam, UN) or cytokeratin 5 (K5, Abcam, United Nations), followed by secondary antibodies from Proteintech.

### 2.6 Estimation of growth factors by enzyme-linked immunosorbent assay (ELISA)

Following 3 days of serum-free cultivation, the cell-free culture medium was harvested and underwent centrifugation. For wound tissue samples, the tissue is homogenized in 10 times the volume of phosphate buffer and the supernatant is collected after centrifugation. The resulting supernatant was utilized for the measurement of growth factors (EGF, bFGF, VEGF, and TGF-β) using the ELISA method (4A Biotech Co., Ltd., China). A Multiskan TM GO spectrophotometer (ThermoFisher, United States) was employed to quantify the absorbance of the plate at a wavelength of 450 nm.

### 2.7 RNA extraction and real-time polymerase chain reaction

The RNA extraction was performed using RNAiso Plus (TaKaRa, Dalian, China). The synthesis of cDNA was accomplished by using a reverse transcriptase and the PrimeScript™ RT reagent kit (TaKaRa, China). Quantification of mRNA levels was performed with SYBR Premix Ex Taq™ (TaKaRa, China) on an Icycler lQ system (Bio-RAD, United States), utilizing the 2^−ΔΔCt^ method for relative gene expression analysis. Primer sequences can be found in Supplementary Table S1.

### 2.8 Knockdown integrin αv in hAMSCs

When hAMSCs reached a confluency of 70%, transfection was conducted using siRNA Transfection Reagent (sc-29528, Santa Cruz). A total of forty picomoles of synthetic small interfering RNAs (siRNAs) were transfected into human amniotic mesenchymal stem cells (hAMSCs) in 24-well plates, targeting either the human integrin subtype αv (sc-29373, Santa Cruz) or a scrambled siRNA (sc-37007, Santa Cruz), following the manufacturer’s instructions. The transfection efficiency was then assessed by RT-PCR after 48 h of continued cultivation. LY294002 (MedChem Express,United States) was used as the inhibitor of PI3K/AKT pathway.

### 2.9 Statistical processing

The statistical analysis was conducted with SPSS version 29.0 (IBM Corp., Armonk, NY, United States), and the results were presented as mean ± standard deviation (x ± s). Normality and homogeneity of variance tests were conducted to assess the distribution and equality of variances, respectively, for all quantitative data. Analysis of variance (ANOVA) was employed to compare multiple groups, while t-tests were performed for parametric comparisons between two groups. A *p*-value of less than 0.05 (*p* < 0.05) was considered statistically significant.

## 3 Results

### 3.1 Peptide hydrogels support hAMSCs survival and proliferation

**FIGURE 1:**
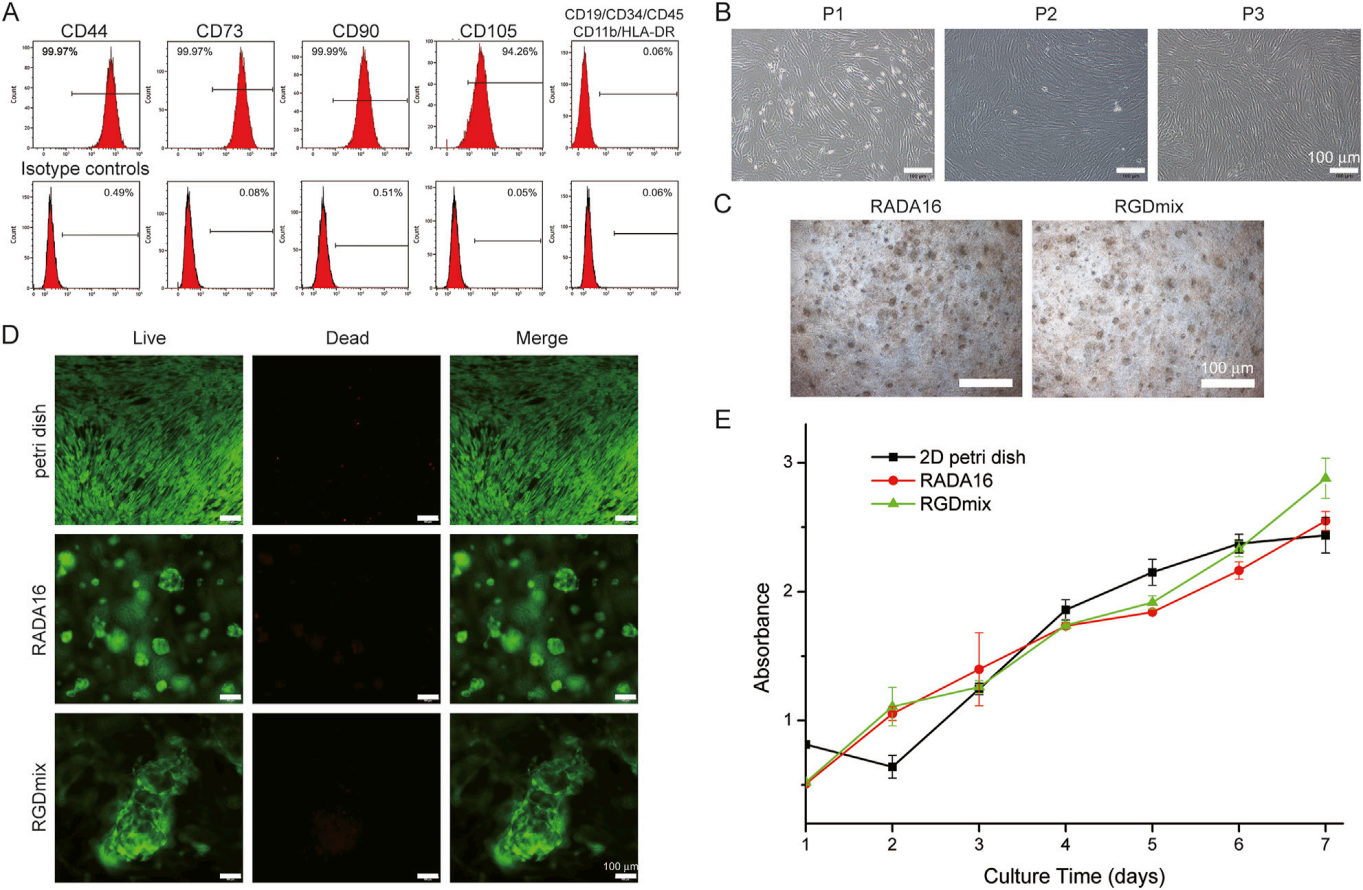
**(A)** Expression of MSCs surface markers: hAMSCs were positive for CD90, CD105, CD73, CD44 markers and negative for CD45, CD34, CD19, CD11b and HLA-DR. **(B)** Morphological characteristics of hAMSCs cultured in petri dishes at the passage 1-3. **(C)** The morphological features of hAMSCs cultured in hydrogels at day 4. **(D)** Live/dead staining of hAMSCs in hydrogels after 4 days of culture. **(E)** Proliferation curves of cultured hAMSCs as determined by the CCK-8 test.

The hAMSCs are a type of mesenchymal stem cell that originate from embryonic mesoderm. In recent years, hAMSCs have gained considerable attention for their potential clinical applications due to their high plasticity, anti-inflammatory properties, and minimal ethical concerns (Naeem et al., 2022). Flow cytometry analysis demonstrated that the extracted hAMSCs expressed high levels of CD44, CD73, CD90, and CD105, while showing no expression of CD45, CD19, CD34, CD11b, and MHC II cell surface molecule (Figure 1A). The trilineage differentiation experiments clearly illustrated the differentiation potential of hAMSCs into osteocytes, chondrocytes, and adipocytes. Moreover, immunohistochemical analysis exhibited strong vimentin expression in hAMSCs, indicating their mesenchymal lineage, while the expression of CK19, an epithelial cell marker, was not detected (Supplementary Figure S1). These characteristics meet the identification criteria for multipotent MSCs established by the International Society for Cellular Therapy (Soliman et al., 2021).

Passages 1–3 of hAMSCs grew rapidly and exhibited a typical spindle-shaped growth and a fibroblast-like phenotype in a swirling arrangement (Figure 1B). When encapsulated in hydrogels, hAMSCs started to proliferate and fuse with each other to form globular cell clusters (Figure 1C). After 4 days of cultivation in the hydrogels, the cell viability was studied using the live/dead staining method (Figure 1D). The results demonstrated that over 95% of the cells embedded in the hydrogel remained alive, indicating the non-toxic nature of the hydrogel and its good compatibility for stem cells survival. Furthermore, the adhesion and proliferation of hAMSCs seeded in RADA16 and RGDmix hydrogels was measured via CCK-8 assays (Supplementary Figure S2; Figure 1E). The results showed that RGDmix hydrogel significantly promoted hAMSC adhesion. Consistent with the live/dead assay, both hydrogels supported rapid cell proliferation. Notably, hAMSCs proliferation decreased due to contact inhibition after 5 days of culture on the 2D petri dish, while the cells in the hydrogels continued to proliferate because the porous structure of the hydrogels effectively extended the growth space for culturing cells.

### 3.2 RGDmix hydrogel improves the secretion of growth factors by hAMSCs *in vitro*

**FIGURE 2:**
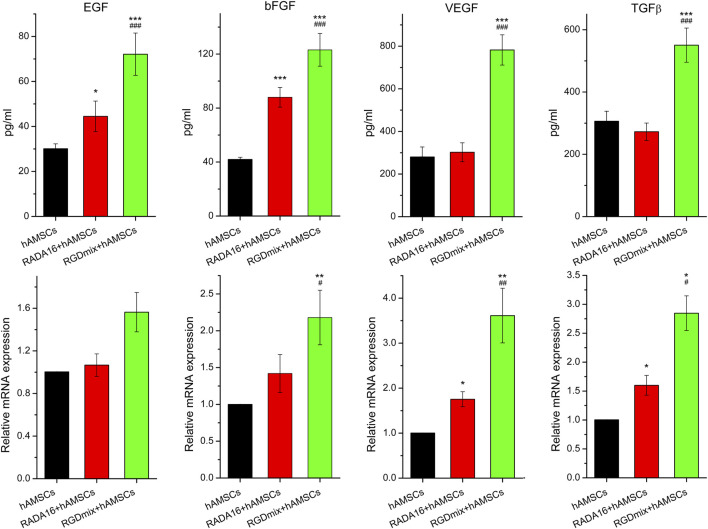
The relative mRNA expression level of EGF、bFGF、VEGF, as well as TGF-β and the amount of protein secretion from hAMSCs encapsulated in hydrogels for 3 days measured by ELISA. The relative mRNA expression was analyzed by the 2^−DDCt^ method, normalized to the Ct of GAPDH and calibrated to hAMSCs treatment groups. Data are expressed as mean ± SD. Significance levels were set at: ^*^ *P*< 0.05, ^**^ *P*< 0.01, ^***^ *P*< 0.001 *vs.* hAMSCs alone; ^#^ *P*< 0.05, ^##^ *P*< 0.01, ^###^ *P*< 0.001 *vs.* RADA16+hAMSCs groups (n = 3).

Soluble growth factors play a promoting role in the process of wound healing by activating specific cell surface receptors. Clinical studies have demonstrated the efficacy of locally administering growth factors in accelerating wound contraction and promoting healing (Yamakawa and Hayashida, 2019). To investigate the impact of the RGDSP motif on growth factor secretion by hAMSCs, we analyzed the gene expression and secretion levels of four growth factors, epidermal growth factor (EGF), basic fibroblast growth factor (bFGF), vascular endothelial growth factor (VEGF) and transforming growth factor-beta (TGF-β). Figure 2 shows that compared to other groups, the mRNA and protein levels of EGF, bFGF, VEGF, and TGF-β were significantly increased when hAMSCs were embedded in RGDmix hydrogel. This indicates that the addition of RGDSP motif enables the RADA16 hydrogel to enhance the expression of multiple growth factors in hAMSCs *in vitro*.

### 3.3 RGDmix hydrogel promotes the wound healing process

Stem cells and hydrogel scaffolds with excellent biocompatibility are key elements in regenerative medicine (Karavasili et al., 2019). Both effectively aid wound healing through various mechanisms. Wound healing is improved by stem cells through multiple mechanisms, including differentiation, immunoregulation, and paracrine signaling (Hu et al., 2020). Hydrogels provide a moist ECM mimic microenvironment, which facilitates the survival, proliferation and migration of cells from wounded areas and prevents wound infection (Guan et al., 2022). Thus the co-delivery of hydrogels and stem cells together can synergistically improve the wound healing.

**FIGURE 3:**
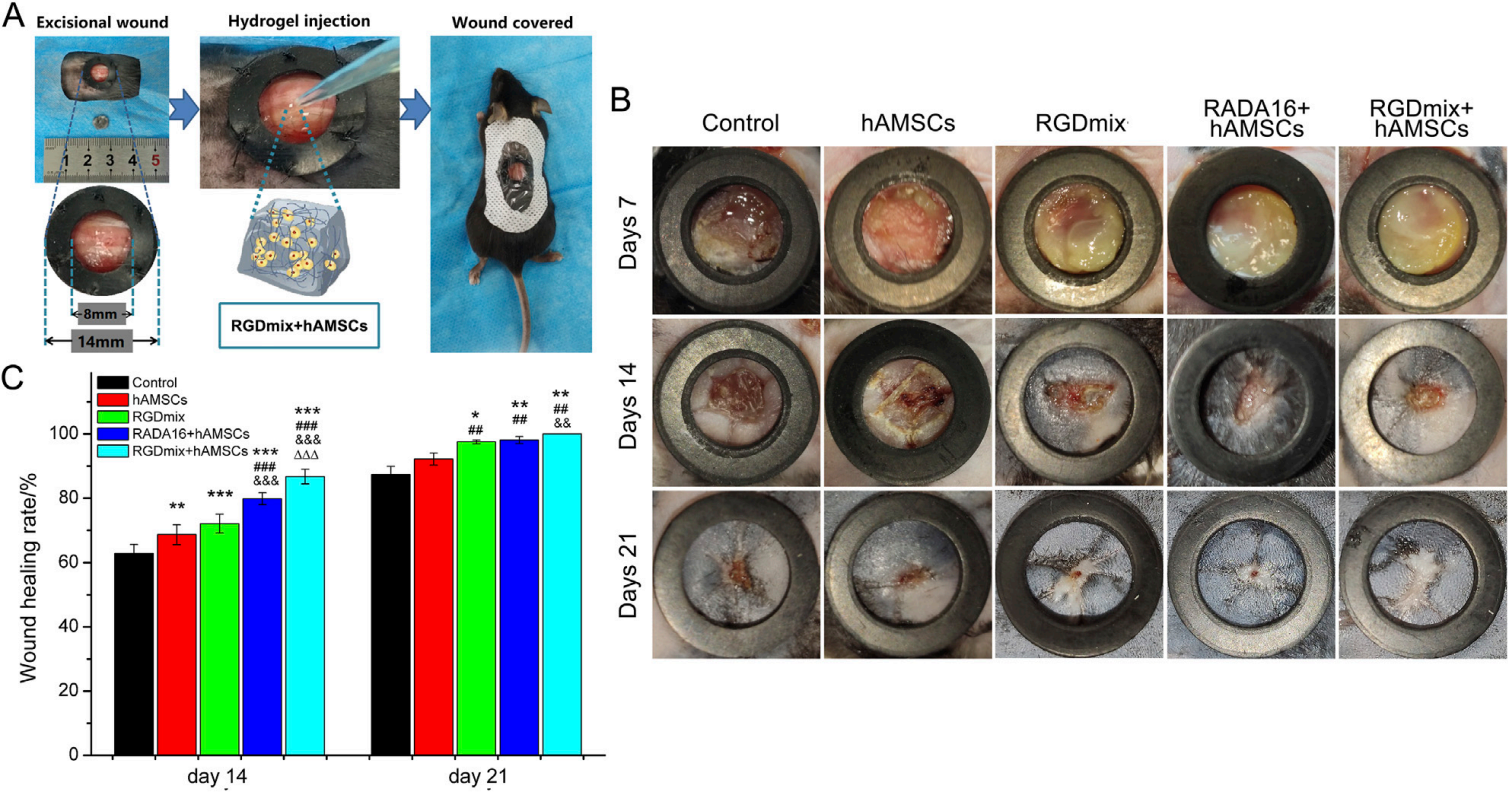
**(A)** Preparation of mouse skin excisional wound model. **(B)** Representative photo images of wounds when treated with different treatments. The inner diameters of the rubber rings used were 8 mm. **(C)** The wound healing rates for the treated wounds were measured and expressed as mean ± SD. Significance levels were set at: ^*^ *P*< 0.05, ^**^ *P*< 0.01, ^***^ *P*< 0.001 *vs.* control; ^##^ *P*< 0.01, ^###^ *P*< 0.001 *vs.* hAMSCs groups; ^&&^ *P*< 0.01, ^&&&^ *P*< 0.001 *vs.* RGDmix groups; ^ΔΔΔ^ *P*< 0.001 *vs.* RADA16+hAMSCs groups (n = 5).

The RGDSP motif, derived from fibronectin, is a cell-adhesive ligand that recognizes multiple integrins and has been shown to effectively promote the survival, proliferation, and phenotypic characterization of hAMSCs (Zhang et al., 2021). In this study, the wound healing-promoting effects of the RGDSP motif were examined in a complete cutaneous trauma model. Wounds were prevented from healing by contraction using a silicone rubber ring firmly attached to the edge of the skin surrounding the wounds (Figure 3A). Figures 3B,C illustrate the healing status of the wounds in each group. Overall, the wound healing rates in the co-transplantation treatment groups using hydrogels and hAMSCs were significantly higher compared to the groups receiving either hAMSCs or RGDmix treatment. On the 7th day, a layer of hydrogel could still be observed on the wound surface of the hydrogel-treated groups, which facilitated wound moisture preservation. By the 14th day, the RGDmix + hAMSCs co-transplantation treatment demonstrated a wound closure rate of 86.7%, which was significantly superior to the other groups, and by the 21st day, the RGDmix + hAMSCs co-transplantation group achieved complete closure of the wound. Surprisingly, there was no significant difference in the wound closure rate between the hAMSCs-treated group and the control group throughout the entire process of wound repair.

The survival of stem cells at the transplantation site is essential for their biological functions to be exerted. In order to monitor the survival of transplanted hAMSCs at the wound site, the Anti-Human Nuclear Antigen antibody (HNA) was utilized. The findings demonstrated that, on the 7th day post-transplantation, hAMSCs were barely detectable at the wounds of the hAMSCs treatment group. Meanwhile, a substantial number of hAMSCs were still observed at the wound sites in the RGDmix + hAMSCs co-transplantation group (Supplementary Figure S3). This observation can be attributed to the peptide hydrogel’s capacity to maintain wound moisture and the RGDSP sequence’s ability to simulate the interaction between stem cells and the extracellular matrix, resulting in significantly enhanced survival of the transplanted hAMSCs.

**FIGURE 4:**
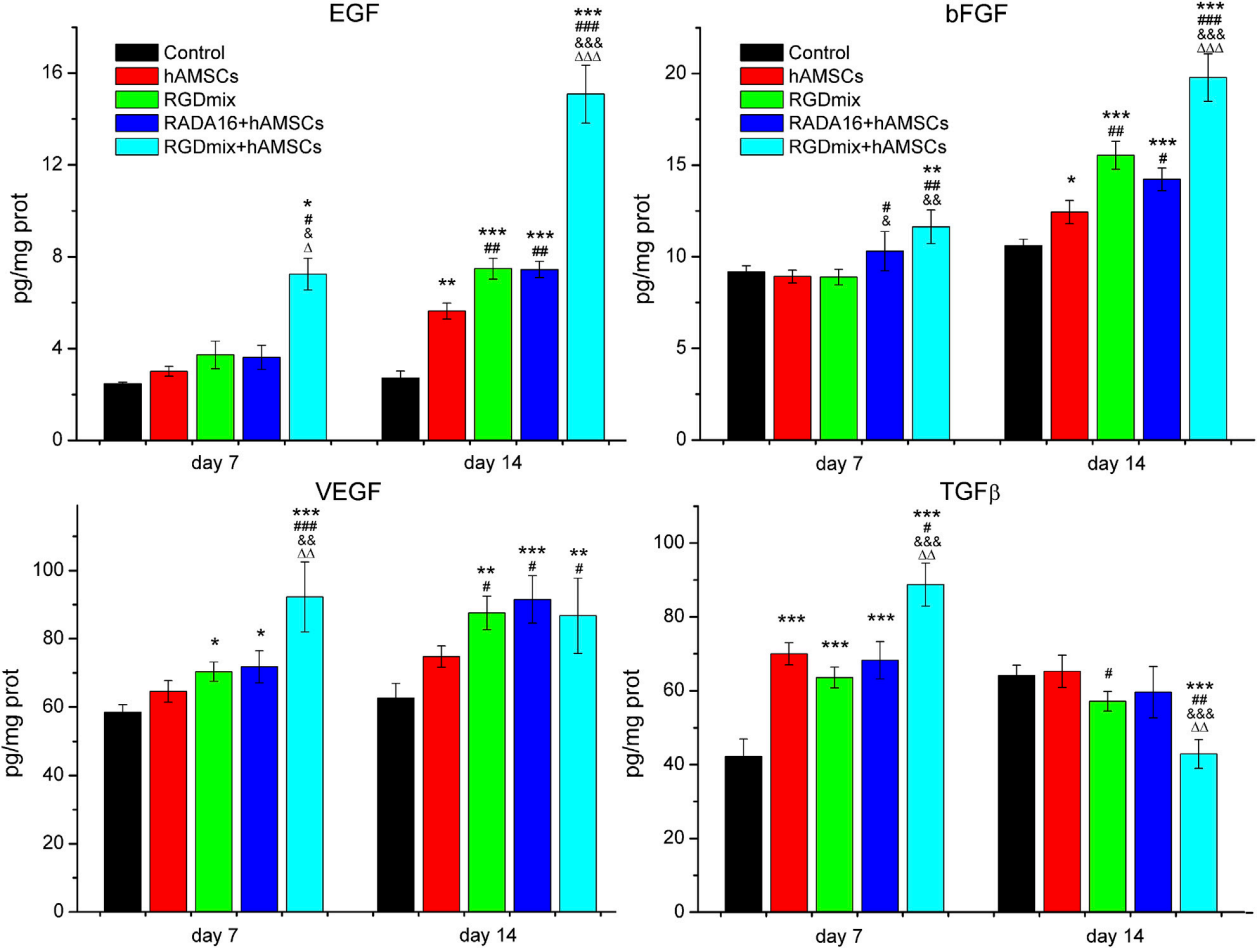
The relative expression levels of EGF、bFGF、VEGF and TGF-β in wounds of each treatment group at day 7 and 14 measured by ELISA. Data are expressed as mean ± SD. Significance levels were set at: ^*^ *P*< 0.05, ^**^ *P*< 0.01, ^***^ *P*< 0.001 *vs.* control; ^#^ *P*< 0.05, ^##^ *P*< 0.01, ^###^ *P*< 0.001 *vs.* hAMSCs groups; ^&^ *P*< 0.05, ^&&^ *P*< 0.01, ^&&&^ *P*< 0.001 *vs.* RGDmix groups; ^Δ^ *P*< 0.05, ^ΔΔ^ *P*< 0.01, ^ΔΔΔ^ *P*< 0.001 *vs.* RADA16+hAMSCs groups (n = 5).

Prolonged survival of transplanted hAMSCs may facilitate the secretion of growth factors by hAMSCs. Thus we further examined the expression levels of EGF, bFGF, VEGF and TGF-β on the 7th and 14th day of the wound healing process using ELISA. As shown in Figure 4, within 14 days, the co-transplantation of RGDmix hydrogel and hAMSCs led to a continuous elevation in the levels of EGF, bFGF, and VEGF in the wound area. These expression levels were markedly greater than those observed in the other groups. Furthermore, the expression levels of TGF-β in the wound area were also increased on the 7th day but rapidly declined on the 14th day. This decline may promote scarless wound healing. In general, the findings suggest that RGDmix hydrogel is beneficial for upregulating the growth factors expression and facilitating wound healing.

**FIGURE 5:**
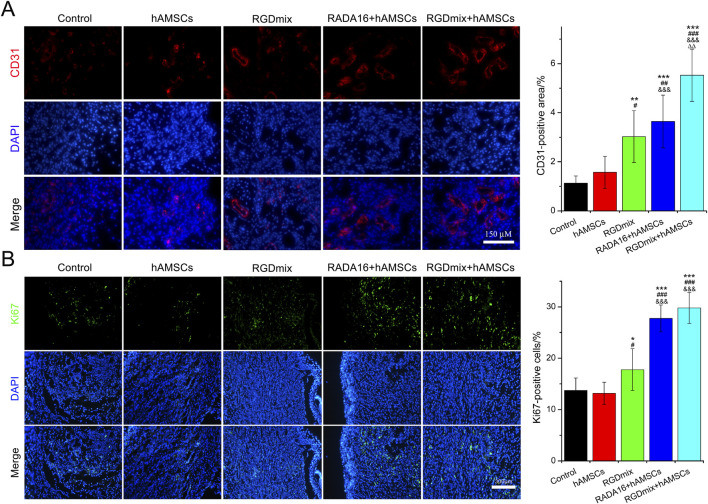
Expression of angiogenesis marker CD31 **(A)** and proliferation marker Ki67 **(B)** at the wound site at day 7. Significance levels were set at: ^*^ *P*< 0.05, ^**^ *P*< 0.01, ^***^ *P*< 0.001 *vs.* control; ^#^ *P*< 0.05, ^##^ *P*< 0.01, ^###^ *P*< 0.001 *vs.* hAMSCs groups; ^&&&^ *P*< 0.001 *vs.* RGDmix groups; ^ΔΔ^ *P*< 0.01 *vs.* RADA16+hAMSCs groups (n = 5).

Angiogenesis and proliferation of epidermal cells are critical for the formation of granulation tissue, tissue remodeling and wound maturation throughout the wound healing process. The formation of new blood vessels enables the supply of supplies nutrients and oxygen to the cells at the wound site and facilitates the entry of immune and reparative cells, thereby accelerating wound healing (Boerckel et al., 2011). Granulation tissue contains a highly organized network of endothelial cells, fibroblasts and immune cells. The proliferation of these cells contributes to ECM synthesis, which is crucial for tissue remodeling (Pang et al., 2017). Thus, the expression of platelet endothelial cell marker CD31 and cell proliferation nuclear antigen Ki67 were analyzed, respectively. As shown in Figure 5 and Supplementary Figure S4, the expression of CD31 in the RGDmix + hAMSCs co-transplantation group on day 7 was significantly greater than that in the other groups. This result may suggest that the RGDmix hydrogels provided an ECM-mimicking scaffold that promotes angiogenesis. Morover, the hAMSCs-loaded hydrogel groups exhibited a notably higher intensity of Ki67-positive cells than the other groups, indicating enhanced cell proliferation resulting in effective tissue remodeling. These results are consistent with the results mentioned above, where hAMSC transplantation alone did not effectively promote angiogenesis, cell proliferation, and wound healing. This could potentially be attributed to the relatively dry environment of the wound (Xu et al., 2023) and the lack of protective effects offered by the hydrogel, resulting in the rapid loss of viability of the transplanted hAMSCs (Wechsler et al., 2021).

### 3.4 RGDmix hydrogel enhances skin regeneration and maturation

**FIGURE 6:**
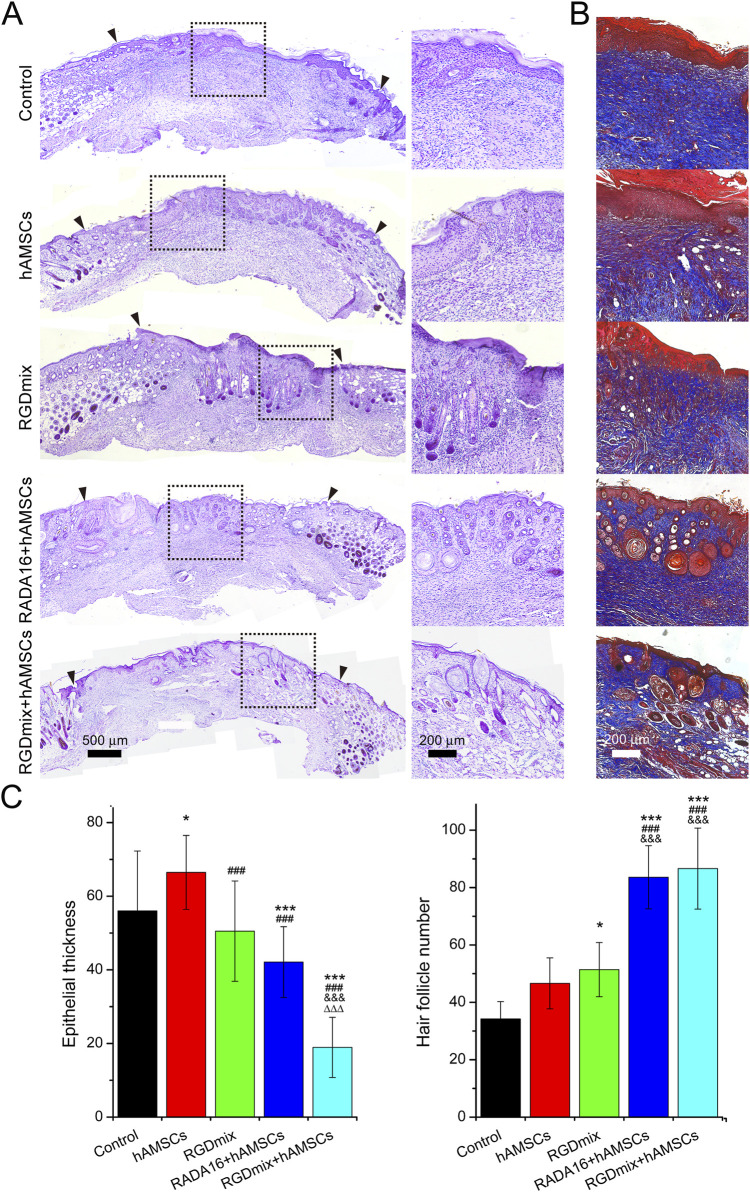
Evaluation of regenerated skin structures at day 21. **(A)** Hematoxylin and eosin or **(B)** Masson’s trichrome stained images of excisional wounds with different treatments. The black arrowheads mark the edges of the original excision sites. Collagen is stained blue. **(C)** Quantification of epidermal thickness and the number of hair follicles. Significance levels were set at: ^*^ *P*< 0.05, ^***^ *P*< 0.001 *vs.* control; ^###^ *P*< 0.001 *vs.* hAMSCs groups; ^&&&^ *P*< 0.001 *vs.* RGDmix groups; ^ΔΔΔ^ *P*< 0.001 *vs.* RADA16+hAMSCs groups (n = 4).

**FIGURE 7:**
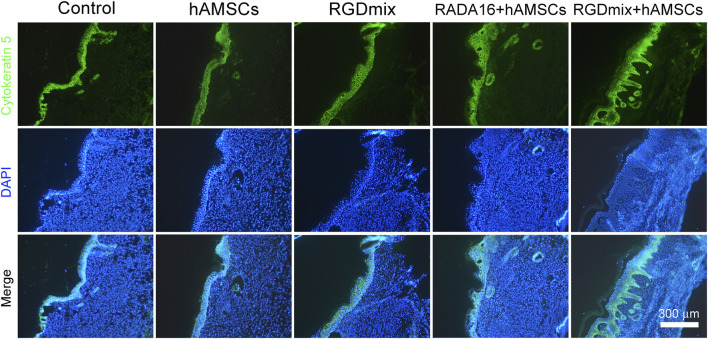
Representative images of K5 immunostaining at day 21. In the RGDmix + hAMSCs co-transplantation treatment group, K5 was predominantly observed in the basal layer, hair follicles, and sebaceous gland regions. In normal skin, K5 expression is limited to the basal layer, the two lowest layers of the stratum spinosum, the outer root sheath of hair follicles, and the myoepithelial cells of sebaceous glands. However, in newborn immature epidermis, epithelial cells maintain a hyperproliferative state, leading to extensive expression of K5 throughout all layers of the stratum spinosum and stratum granulosum.

Restoration of skin structure and function is the ultimate goal of wound repair. We further investigated the structure of regenerating epidermis in the late stage of wound healing. As shown in Figure 6, all wounds underwent re-epithelialization and formed thick granulation tissue with dense collagen deposition. Importantly, epidermal tissue with stratum corneum, stratum granulosum, and stratum spinosum, which provide essential barrier function, also formed. However, in the two groups treated with hAMSCs-loaded hydrogels, a greater number of hair follicles and sebaceous gland structures were observed in the newly formed epidermis, particularly near the original excision sites. In contrast, the RGDmix treatment group demonstrated a significant reduction in hair follicle regeneration, whereas both the control and hAMSCs-treated groups displayed minimal hair follicle regeneration. Quantitative analysis of epidermal thickness revealed varying degrees of cell proliferation in all wounds, with a thinner epidermis observed near the original excision sites in comparison to the central region of the wound. However, in the wounds subjected to the RGDmix + hAMSCs construct, the regenerated epidermis was denser and significantly thinner than in the other groups, approaching the thickness of normal mouse skin (8.8 ± 4.2 μm), as measured in this study. Consistent with this data, immunofluorescent staining of cytokeratin 5 (K5) in sections of regenerating skin revealed that K5 expression was primarily observed in the basal layer of the regenerated epidermis, the lowest few cell layers of the stratum spinosum, and in the hair follicles and sebaceous glands in the wounds treated with the RGDmix + hAMSCs construct, with minimal labeling in the stratum granulosum (Figure 7). The pattern of K5 protein expression closely resembles that of the normal epidermis in mice (Doukas et al., 2021). In contrast, every layer of the epidermis in the regenerated epidermis of the other groups showed labeling with the K5 antibody. These results indicate that RGDmix hydrogel enhances epidermis regeneration and maturation, improving the quality of wound healing.

### 3.5 RGDmix enhances paracrine function of hAMSCs via integrin αv/PI3K/AKT axis

**FIGURE 8:**
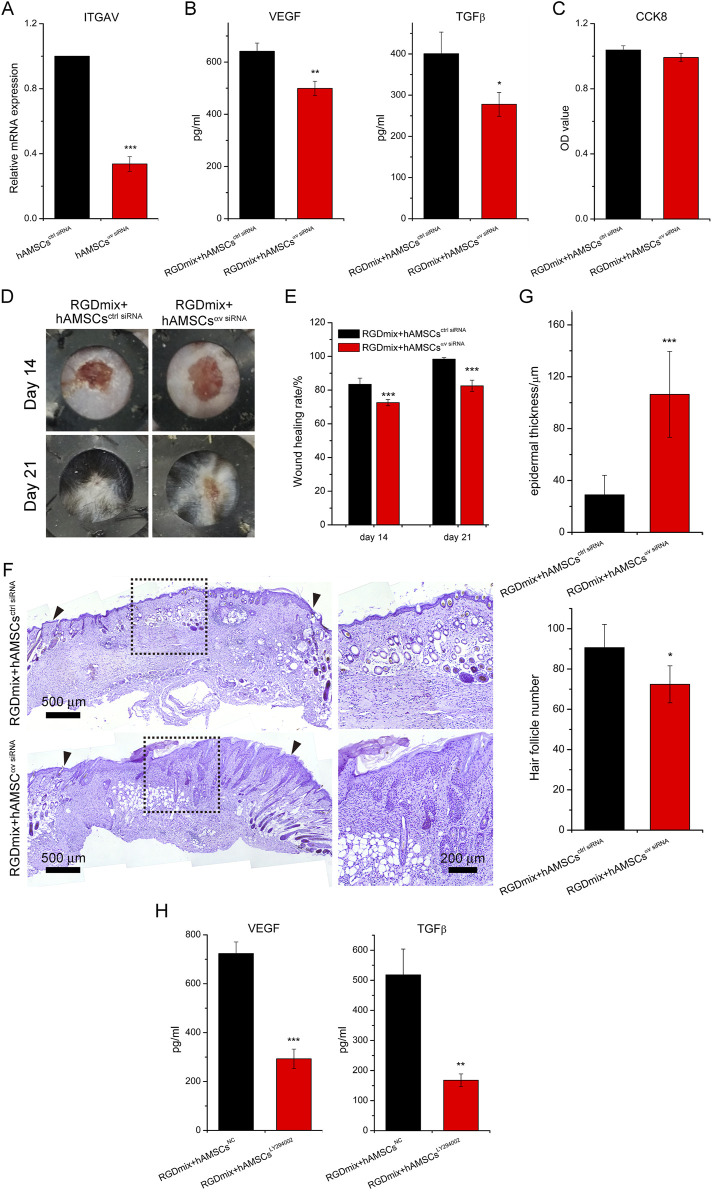
RGDmix hydrogel enhances the secretion function of hAMSCs through the integrin αv/PI3K/AKT axis. **(A)** qRT-PCR confirmed the knockdown of integrin αv. **(B)** ELISA experiments showed that silencing integrin αv significantly reduces the expression levels of growth factors VEGF and TGF-β in hAMSCs encapsulated in RGDmix hydrogel. **(C)** CCK-8 assay demonstrated that knockdown of integrin αv does not affect the proliferation of hAMSCs. **(D)** Representative photo images, **(E)** wound healing rates, and **(F)** Hematoxylin and eosin stained images of excisional wounds treated with different treatments. The inner diameters of the rubber rings were 8 mm. The blackarrow heads mark the edges of the original excision sites. **(G)** Quantification of epidermal thickness and the number of hair follicles (n = 5). **(H)** Expression levels of growth factors VEGF and TGF-β in hAMSCs encapsulated in RGDmix hydrogel when the PI3K/AKT pathway was inhibited by LY294002 (n = 3). Significance levels were set at: ^*^ *P*< 0.05, ^**^ *P*< 0.01, ^***^ *P*< 0.001 *vs.* control.

Our previous study found that encapsulation of hAMSCs in RGDmix hydrogel increased the mRNA expression levels of the integrin αv (Zhang et al., 2021). To verify the regulatory role of the integrin αv subunit on the expression levels of growth factors in hAMSCs, the integrin αv expression in hAMSCs was silenced (Figure 8A), and the levels of VEGF and TGF-β were subsequently detected (Figure 8B). The results showed that silencing the integrin αv gene resulted in reduced levels of VEGF and TGF-β expression. Furthermore, CCK-8 experiments demonstrated that silencing the integrin αv gene did not affect the viability and proliferation of hAMSCs (Figure 8C). This suggests that the increased concentration of growth factors in the medium is attributed to the enhanced paracrine function of hAMSCs mediated by integrin αv, rather than the proliferation of hAMSCs. To further confirm the function of integrin αv in wound healing, which mediates the association between hAMSCs and RGDmix hydrogel scaffold, we compared the therapeutic effect of hAMSCs with or without integrin αv silencing on full-thickness skin wound healing when co-transplanted with RGDmix hydrogel. The findings indicated that the silencing of integrin αv in hAMSCs significantly delayed the wound healing (Figures 8D,E). HE staining showed a reduction in the formation of new skin follicles, as well as a significant rise in the thickness of the hyperplastic epidermis (Figures 8F,G), indicating relative low maturity of the newly formed epidermis. Moreover, the levels of VEGF and TGF-β expression were assessed in the presence of LY294002, a specific inhibitor of the PI3K/AKT pathway. This analysis aimed to further explore the involvement of the PI3K/AKT pathway in elevating the secretory capacity of growth factors in hAMSCs (Figure 8H). The results showed that even when embedded in RGDmix hydrogel, inhibition of the PI3K/AKT pathway still significantly decreased the expression levels of VEGF and TGF-β. Collectively, these findings demonstrate that RGDmix hydrogel enhances the secretion of growth factors by hAMSCs via the PI3K/AKT pathway. Moreover, the findings of this study demonstrate that activation of the RGDSP/integrin αv/PI3K/AKT axis can stimulate the paracrine function of hAMSCs and facilitate wound healing.

## 4 Discussion

Stem cell-based therapy is currently considered to be one of the most promising approaches in the area of regenerative medicine and can be used to treat a variety of conditions, including graft versus host disease, neurodegenerative diseases, bone and cartilage regeneration, and wound repair (Matsuzaka and Yashiro, 2022). It has been documented that the therapeutic advantages observed subsequent to the transplantation of stem cells are mainly derived from their paracrine function, instead of their capacity for differentiation (Brennan et al., 2020). Stem cells have the remarkable ability to secrete various soluble cytokines, including growth factors. Recent investigations have unveiled that enhancing the paracrine function of stem cells can significantly increase their therapeutic effect (Liu et al., 2022). However, after stem cells are transplanted into the damaged area, the majority of them quickly lose their activity and are ultimately cleared due to local oxidative stress, inflammation reactions, inadequate environmental support, and insufficient oxygen and nutrient supply (Brennan et al., 2020). Therefore, improving the survival and secretion function of transplanted stem cells can help to enhance the outcomes of wound healing (Lagneau et al., 2023).

Stem cells reside in the ECM, which provides numerous biophysical signals to support their survival and regulate their fate. Integrins are the major class of cell surface receptors through which stem cells adhere to the ECM, initiating intracellular signaling pathways that enable them to sense mechanical cues from the ECM and respond accordingly (Pang et al., 2023). Therefore, using cell-adhesive peptides to simulate the ECM-stem cell interaction can effectively increase stem cell survival at the transplantation site and enhance their secretion capacity, thus improving the therapeutic effect (Lagneau et al., 2023; Wechsler et al., 2021). For example, the YIGSRGD peptide hydrogel, which combines YIGSR and RGD motifs, can enhance the secretome of MSCs through integrin α2β1, thus improving the therapeutic effect on acute kidney injury (Han et al., 2022). PEG based Microporous annealed particle hydrogel functionalized with integrin α5β1-specific sequence RGDS and c (RRETAWA) can enhance adhesion, spreading, and proliferation of human MSCs (hMSCs), and increase the expression of VEGF and bone morphogenetic protein-2 (Zhang et al., 2020). Similarly, PEG gel modified with GFOGER, a peptide targeting α2β1 integrin, can regulate the adhesion, osteogenic differentiation, and secretion function of hMSCs, and improve their repair capacity by improving their survival and reparative activity at the site of mouse bone defects (Clark et al., 2020).

hAMSCs, which are mesenchymal stem cells obtained from the amniotic membrane, represent a promising source of stem cells for cell therapy. These cells offer several advantages, including ease of procurement, high plasticity, ultra-low immunogenicity, and low tumorigenicity (Zhang et al., 2021). Furthermore, their application does not pose significant medical ethical or legal concerns, thereby making hAMSCs an attractive option for clinical applications (Rady et al., 2020; You et al., 2020).

Previous studies have shown that incorporating adhesive peptides such as RGDSP favors the maintenance of phenotypic characterization, promotes the pluripotent markers expression in hAMSCs, and contributes to the expression of integrin αv and α5 (Zhang et al., 2021). However, the effect of these peptides on growth factor secretion by hAMSCs, wound healing efficiency promotion, and promotion of epidermal maturation, as well as the underlying mechanisms, have yet to be explored.

Herein, we observed that RGDmix effectively upregulated the mRNA and protein levels of EGF, bFGF, VEGF, and TGF-β, *in vitro* (Figure 2). Co-transplantation of RGDmix and hAMSCs also significantly elevated the expression levels of the growth factors within the injury (Figure 4). EGF serves to stimulate the proliferation and migration of fibroblasts and keratinocytes by binding to the EGF receptor, thereby accelerating wound contraction and inducing dermal regeneration and maturation. bFGF facilitates the migration, proliferation, and differentiation of various cells, especially keratinocytes and fibroblasts, promoting granulation tissue formation, wound reepithelialization, ECM remodeling and neovascularization. VEGF governs vascular permeability and orchestrates the recruitment of various inflammatory cells to the site of injury. Simultaneously, it promotes the proliferation and migration of endothelial cells, triggers angiogenesis, and facilitates the reepithelialization of wounds (Pang et al., 2017). Therefore, the upregulation of EGF, bFGF, and VEGF is beneficial for wound healing. TGF-β, on the other hand, is a cytokine secreted by various cells including platelets, macrophages, neutrophils, and fibroblasts, and can regulate fibroblast and inflammatory cell chemotaxis to the wound site through the Smad pathway. TGF-β can promote wound neovascularization, regulate granulation tissue formation and ECM deposition. However, overexpression of TGF-β during tissue remodeling phase can lead to activated keratinocyte proliferation, excessive collagen deposition, and scar formation (Wang et al., 2022). Thus, modulating TGF-β expression is crucial for rapid and scar-free wound healing. On the 7th day, the content of TGF-β in the wounds treated with RGDmix hydrogel and hAMSCs rapidly increased and was significantly higher than in the other groups. This promotes the effective recruitment of fibroblasts in the early stage of wound healing, facilitating the formation of granulation tissue and deposition of ECM. Meanwhile, on the 16th day of treatment, the concentration of TGF-β dramatically decreased, indicating that RGDmix hydrogel may facilitate scarless wound healing by inhibiting the production of TGF-β. Thus, RGDSP-modified hydrogels can better regulate the expression of growth factors in hAMSCs and accelerate wound repair.

Importantly, when combined with hAMSCs, RGDmix hydrogel not only significantly accelerated wound healing, but also improved epidermis regeneration and maturation (Figures 6, 7). RGDmix hydrogel promoted the regeneration of appendages such as hair follicles and sebaceous glands in the newly formed epidermis at the wound site. In fact, in the treatment of cutaneous injuries, the regeneration of hair follicles and other appendages is a bottleneck for the complete restoration of skin structure and function (Huang et al., 2016). In mice, normal epidermis consists of only 2-3 cell layers. In pathological conditions or when subject to trauma, excessive proliferation of keratinocytes and epidermal cells leads to hyperplastic epidermal layers. After wound repair is completed, the newly regenerated hyperproliferative epidermis undergoes remodeling and gradually restores to the normal thickness of the epidermis. Surprisingly, the newly formed epidermis in wounds treated with RGDmix hydrogel and hAMSCs had undergone remodeling, resulting in a thickness of 18.9 ± 8.2 μm. This measurement is very close to the thickness of normal epidermis in mice measured under our conditions (8.8 ± 4.2 μm). In contrast, the newly formed epidermis in the other groups exhibited considerable hyperplasia, with increased stratification and thickness compared to the normal epidermis. Similar results were also observed in K5 immunofluorescence staining. In wounds treated with RGDmix hydrogel and hAMSCs, the expression of K5 was mainly restricted to the basal layer and lower stratum spinosum. However, in other groups, K5 was extensively expressed in all layers of the regenerating epidermis, including the basal layer, spinous layer, and granular layer. K5 functions as a cytoskeletal protein critical for the maintenance of cell morphology and mechanical strength. Studies have shown that in normal epidermis, K5 is mainly expressed in the basal layer of the epidermis, the hair follicles, and the lower two layers of the stratum spinosum. However, in pathological conditions or immature regenerated epidermis, K5 is widely expressed in the epidermis (Doukas et al., 2021; Eylert et al., 2021). Therefore, these results suggest that after 21 days of combined treatment with RGDmix hydrogel and hAMSCs, wounds in mice exhibit a more mature epidermal morphology. In conclusion, RGDmix hydrogel further enhances wound repair and promotes epidermis regeneration and maturation.

Each integrin heterodimer in mammals consists of α and β subunits. Currently, it has been discovered that 18 α subunits and 8 β subunits collectively assemble into 24 distinct integrin receptors. The extracellular binding of integrins promotes the clustering of integrins and the formation of integrin adhesion complexes. This then leads to the reinforcement of the association with the cytoskeleton and the activation of downstream signal transduction through various protein tyrosine kinases, such as focal adhesion kinase and Src family kinases (Chastney et al., 2021). This triggers various cellular signaling events, spanning from cell adhesion, survival, proliferation, motility, gene expression, and differentiation. Among them, the integrin αv subunit can form various RGD-binding integrins with β1, β3, β5, β6, and β8 subunits, which interact with numerous proteins such as vitronectin, fibronectin, collagen, thrombospondin, E-cadherin, vascular cell adhesion molecule-1, VEGF, and TGF-β in the ECM (Chastney et al., 2021). These ligand binding and force application initiate downstream signaling, including the PI3K/AKT pathway, thereby improving tissue regeneration, epidermal generation, and ECM remodeling and angiogenesis (Dhavalikar et al., 2020). Integrin αv is upregulated during wound healing (Duperret et al., 2016). We have previously confirmed that RGDmix can upregulate the expression of the integrin αv subunit in hAMSCs (Zhang et al., 2021). Studies have reported that PEG gels incorporating the RGD sequence can enhance the survival and integrin αv expression in hMSCs, promoting bone repair (Clark et al., 2020). Soft silk hydrogel can upregulate the expression of integrin αv and α2 in umbilical cord mesenchymal stem cells for the treatment of acute kidney injury (Phuagkhaopong et al., 2021). Taken together, we hypothesize that integrin αv may be a key mediator in RGDmix hydrogel’s ability to enhance growth factors expression in hAMSCs. To validate our hypothesis, the integrin αv was silenced, and the secretion levels of VEGF and TGFβ in hAMSCs were detected. As shown in Figures 8A–C, silencing of the integrin αv led to a significant reduction in the secretion levels of VEGF and TGFβ. Meanwhile, a CCK-8 experiment indicated that silencing of the integrin αv did not significantly impact the proliferative activity of hAMSCs. Our data suggest that the enhanced expression of VEGF and TGFβ in hAMSCs embedded in RGDmix hydrogel is attributed to the enhancement of the secretory function rather than an increase in cell number. Furthermore, *in vivo* experiments confirmed that silencing of the integrin αv in hAMSCs indeed reduced the therapeutic effect on wound healing (Figures 8F, G). Furthermore, it is worth noting that the integrin αv can interact with various integrin β subunits to form different integrin complexes. Therefore, further in-depth studies are needed to identify the specific β subunits involved under our conditions.

The PI3K/AKT pathway plays an important role in wound healing by enhancing cell migration, proliferation, angiogenesis, motility, and metabolism (Wei et al., 2020). It regulates the expression of growth factors including EGF, bFGF, VEGF, and TGF-β (Jere et al., 2019). These growth factors can also activate the PI3K/AKT pathway via the stimulation of receptor tyrosine kinases (Zheng et al., 2022). Furthermore, the stimulation of the PI3K/AKT pathway is influenced by the integration of mechanical and biochemical signals from the ECM through integrin αv (Pang et al., 2023; Wang et al., 2020). To investigate the role of the PI3K/AKT pathway in promoting the paracrine function of hAMSCs, the classic inhibitor LY294002 was used. Results showed that LY294002 inhibited the phosphorylation of AKT and mTOR, leading to a blockade of the PI3K/AKT pathway. This blockade led to a significant decrease in the levels of VEGF and TGFβ in hAMSCs, even when they were embedded in RGDmix hydrogel. This suggests that the RGDmix hydrogel promotes the growth factors expression in hAMSCs through the PI3K/AKT pathway. Interestingly, the decrease in the levels of VEGF and TGFβ in the LY294002 inhibition experiment was greater than that in the silencing experiment of integrin αv, indicating the potential involvement of alternative pathways associated with integrin αv in the regulation of growth factor expression in hAMSCs. Firstly, the RGDSP sequence can bind to integrins containing other α subunits, such as α5 and α8 subunits, in addition to the αv subunit. Secondly, the activation of the PI3K/AKT pathway may also be related to the stiffness of the hydrogel. The mechanical properties of the ECM affect cellular tension and intracellular signaling pathways (Kulkarni et al., 2023). Some studies have shown that soft nanofiber hydrogels can activate the PI3K/AKT pathway through different mechanisms. For example, soft hyaluronic acid can activate the PI3K/AKT pathway, promoting the extension, focal adhesion, and membrane tension of murine fibroblasts 3T3 cells and hepatocellular carcinoma cells (Huh7) (Mandal et al., 2020). A suitable “soft” environment can trigger angiogenic and osteogenic signals of bone marrow mesenchymal stem cells via the focal adhesion pathway and the downstream PI3K/AKT pathway (Ma et al., 2021). Gelatin methacryloyl (GelMA) substrate with relatively low hardness, as well as Matrigel and Collagen I composite gels can promote the gene expression and pathway activation of the PI3K/AKT pathway in vascular endothelial cells and vascular smooth muscle cells (Li et al., 2022; Zheng et al., 2022). In our work, the functionalized RGDSP sequence significantly influences the self-assembly process of short peptides and hydrogel formation, resulting in a weaker hydrogel (∼600 Pa) (Zhang et al., 2021), compared to RADA16. Thus, the lower mechanical strength of RGDmix may also contribute to the PI3K/AKT pathway activation. Collectively, the incorporation of the RGDSP sequence and the compliant stiffness of the hydrogel may synergistically affect the expression of growth factors in hAMSCs.

In conclusion, our results confirm that the incorporation of the cell adhesion sequence RGDSP in peptide hydrogels effectively promotes the expression of various growth factors in hAMSCs through the activation of the integrin αv-mediated PI3K/AKT pathway, thereby enhancing wound healing and epidermal maturation. Our findings suggest that the design of novel biomaterials to mimic ECM components can improve the external microenvironment of MSCs, increase their cytokine secretion capacity, and regulate their fate, which may offer valuable insights for the development of MSCs-based cell therapies in regenerative medicine.

## Data Availability

The datasets presented in this study can be found in online repositories. The names of the repository/repositories and accession number(s) can be found in the article/Supplementary Material.
