# Supplementary material for: RGDSP-functionalized peptide hydrogel stimulates growth factor secretion via integrin αv/PI3K/AKT axis for improved wound healing by human amniotic mesenchymal stem cells
Source: Front Bioeng Biotechnol. 2024 Oct 14;12:1385931. doi: 10.3389/fbioe.2024.1385931 (PMC11513332; doi:10.3389/fbioe.2024.1385931)
Supplement: Supplementary file 1 [file DataSheet1.docx]

Supplementary Material

Table S1 The primer sequences and annealing temperatures

| **Gene** | **Genebank number** | **Sequences(5’→ 3’)** | **Temp**  **(℃)** |
| --- | --- | --- | --- |
| PECAM1 | [NM_001032378.2](https://www.ncbi.nlm.nih.gov/nuccore/NM_001032378.2) | For: ACCGGGTGCTGTTCTATAAGG | 61.3 |
|  |  | Rev: TCACCTCGTACTCAATCGTGG | 61.2 |
| VEGFA | NM_001025366.3 | For: AGGGCAGAATCATCACGAAGT | 61.2 |
|  |  | Rev: AGGGTCTCGATTGGATGGCA | 62.9 |
| TGFB1 | NM_000660.7 | For: CAATTCCTGGCGATACCTCAG | 60.2 |
|  |  | Rev: GCACAACTCCGGTGACATCAA | 62.9 |
| FGF2 | NM_002006.6 | For: AGTGTGTGCTAACCGTTACCT | 61.1 |
|  |  | Rev: ACTGCCCAGTTCGTTTCAGTG | 62.8 |
| EGF | NM_001963.6 | For: TGTCCACGCAATGTGTCTGAA | 62.2 |
|  |  | Rev: CATTATCGGGTGAGGAACAACC | 60.7 |
| GAPDH | NM_002046.7 | For: TCAAGAAGGTGGTGAAGCAGG | 61.3 |
|  |  | Rev: AGCGTCAAAGGTGGAGGAGTG | 60.9 |
| ITGAV | NM_002210.5 | For: ATCTGTGAGGTCGAAACAGGA | 60.5 |
|  |  | Rev: TGGAGCATACTCAACAGTCTTTG | 60.2 |


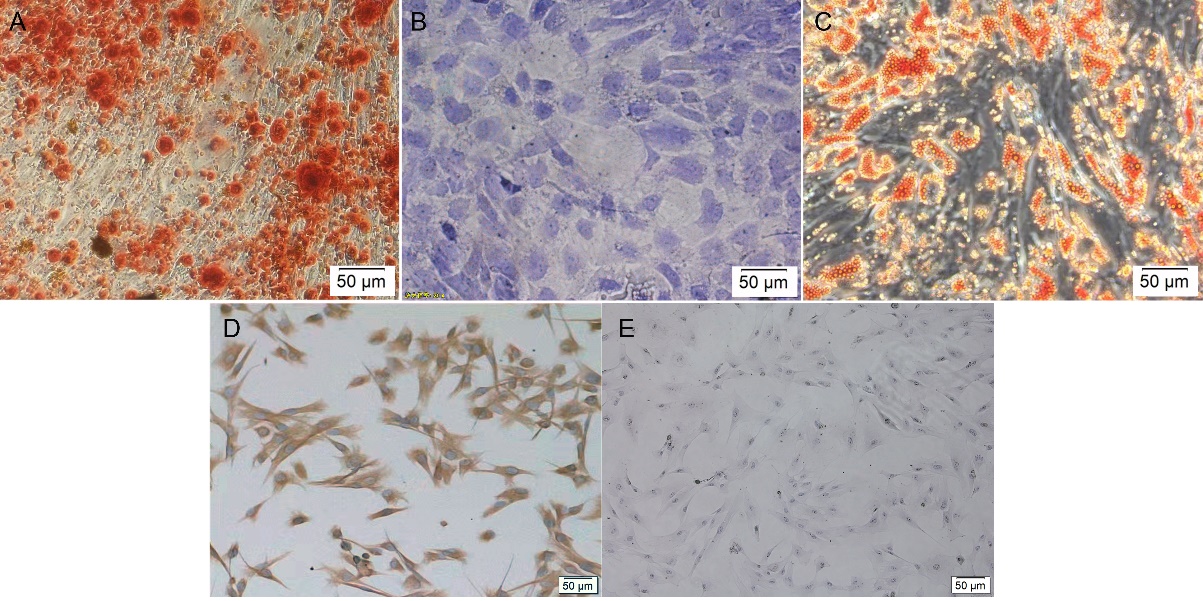


**Figure S1.** Characterization of hAMSCs: hAMSCs induced by osteogenic (A), chondrogenic (B), and adipogenic (C) media were stained using Alizarin Red S, Toluidine Blue solution, and Oil Red O, respectively. Expression of cytoskeletal proteins Vimentin (C) and CK19 (D) in hAMSCs.

.


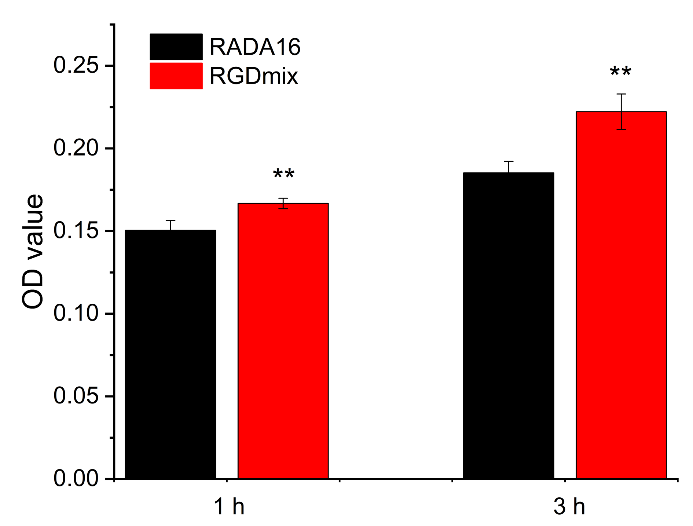


**Figure S2.** Comparison of hAMSCs adhesion on RADA16 and RGDmix hydrogels after PBS rinses at 1 h and 3 h. Data are expressed as mean ± SD. Significance levels were set at: **P< 0.01 (n = 5).


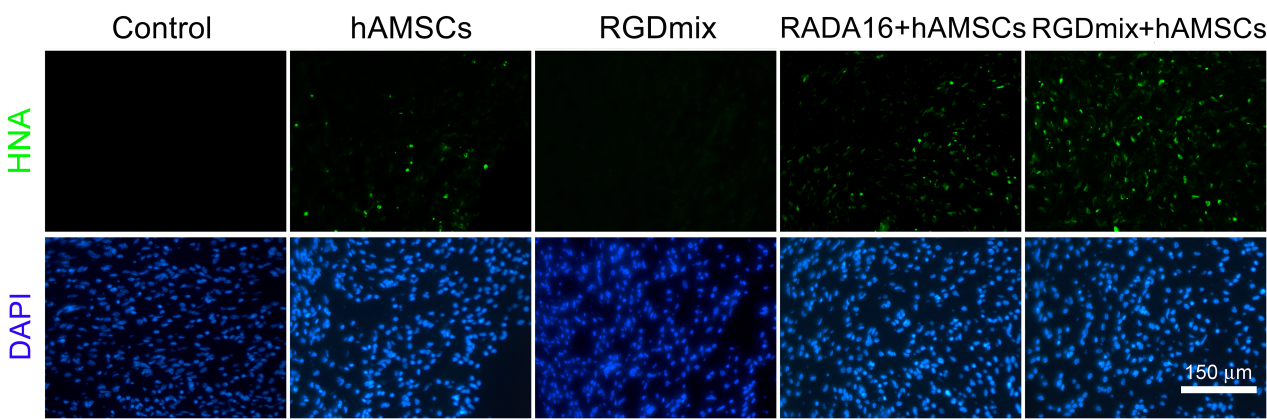


**Figure S3.**  Immunofluorescence staining for identification of hAMSCs using HNA antibody counterstained with DAPI for nuclear staining on day 7.





**Figure S4.** The relative expression levels of CD31 in wounds of each treatment group at day 7 and 14 measured by RT-PCR. Data are expressed as mean ± SD. Significance levels were set at: **P< 0.01, ***P< 0.001 vs. control; ^##^*P*< 0.01, ^###^*P*< 0.001 *vs.* hAMSCs groups; ^&&^P< 0.01 vs. RGDmix groups; ^ΔΔΔ^*P*< 0.001 *vs.* RADA16+hAMSCs groups (n = 5).

**
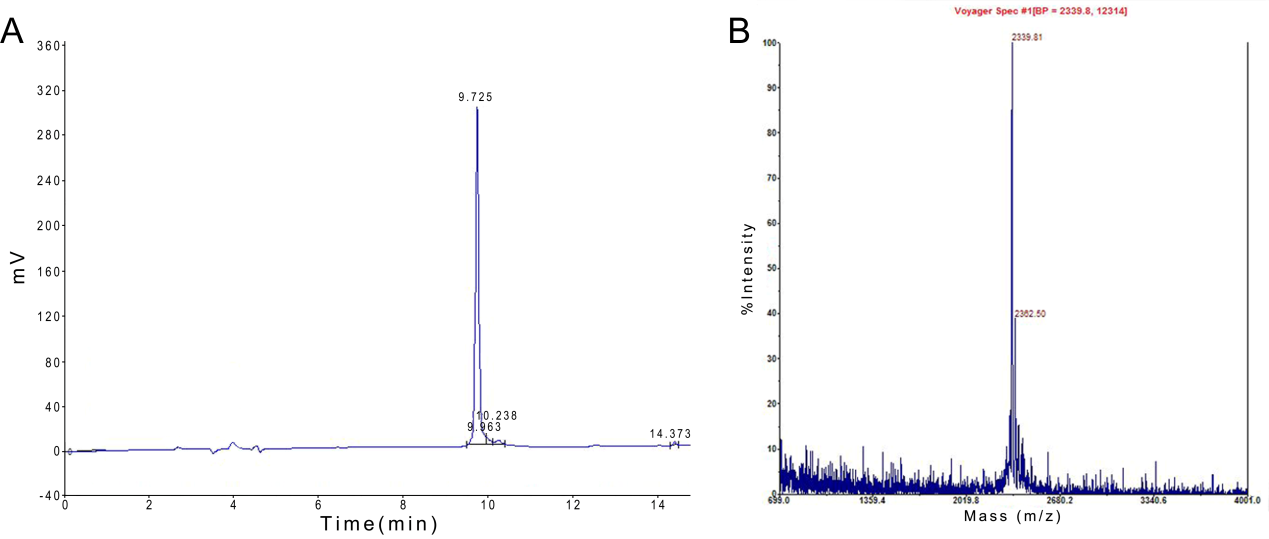
**

**Figure S5.** (A) HPLC chromatogram and (B) MALDI-TOF MS of peptide RADA16-RGDSP.


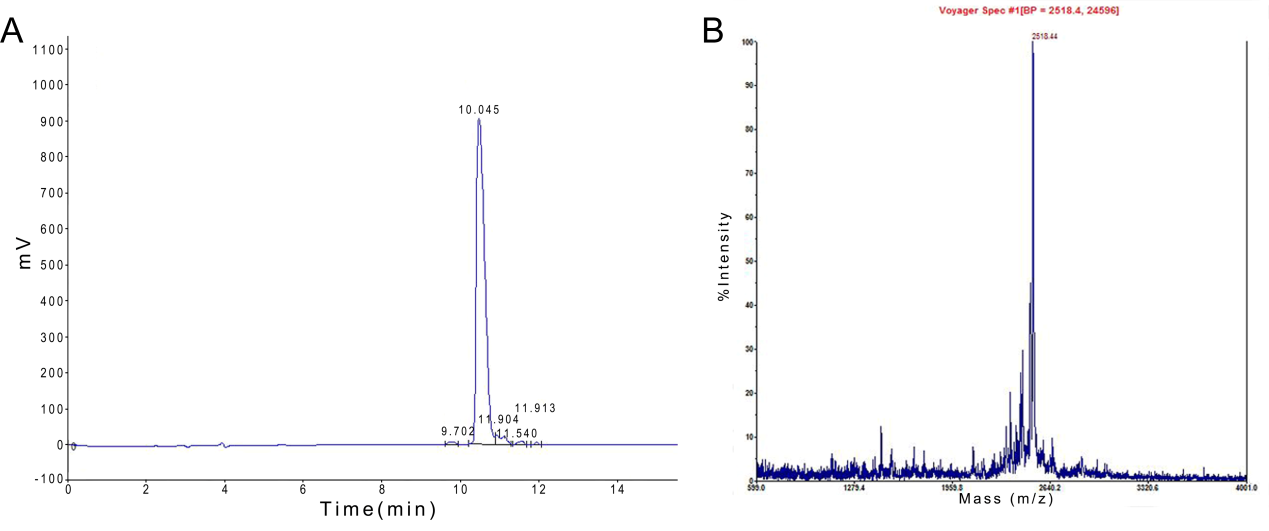


**Figure S6.** (A) HPLC chromatogram and (B) MALDI-TOF MS of peptide RADA16.
